# Motor ability and working memory in Omani and German primary school-aged children

**DOI:** 10.1371/journal.pone.0209848

**Published:** 2019-01-14

**Authors:** Petra Jansen, Clara Scheer, Kashef Zayed

**Affiliations:** 1 Faculty of Psychology, Pedagogic and Sport Science, University of Regensburg, Regensburg, Germany; 2 Department of Physical Education, Sultan Qaboos University, Muscat, Sultanate of Oman; Newcastle University, UNITED KINGDOM

## Abstract

This study investigated the motor ability and working memory performance of Omani and German primary school-aged children. One hundred eighty-five children from public schools participated in a gross motor test that integrated whole body coordination, three different ball tasks, and a 20-meter run. Furthermore, they completed four working memory tests (the Digit-Span Test forward and backwards and the Corsi Block-Tapping Test forward and backwards). Two MANOVAS with the different motor and working memory tests and one univariate analysis of the general motor ability with the between-subject factors group and gender were conducted. Additionally, correlations between motor ability and working memory scores were executed. German children outperformed Omani children in the overall measurement of motor ability, (p = .01) and all aspects of working memory, (all p< .015). There were no correlations between motor and cognitive variables, when analyzing the results for the Omani and German children separately. These findings may be a result of different educational styles or socioeconomic status and must be investigated in more detail.

## Introduction

It is the main goal of this study to investigate the motor ability and working memory performance of Omani and German primary school-aged children. Further, the possible relationship between both aspects will be examined.

The investigation of motor ability is very manifold due to the multifarious differentiations: One possible differentiation is the one in gross motor skill, fine motor skill, bilateral body coordination and timed performance in movement. Physical ability, such as strength, agility, flexibility and balance, are included in in the category of gross motor skills [1]. Few studies have investigated cultural differences in motor ability. One study that compared primary school-aged children in Cameroon and Germany indicated that Cameroonian children have a better motor ability than German children. A reason for this finding might be the early motor stimulation, which differs in both countries [2]. Similarly, Belgian children scored higher in motoric tasks than Australian children, which is explained by their reduced physical activity [3]. Although Chinese children outperformed American children in manual dexterity and balance, American children performed better in a throwing and catching task [4]. These disparities are explained by different cultural practices.

Concerning the gender gap in motor ability in children, a recently published study indicated a difference between 5- to 11-year-old boys and girls: girls performed better in a balancing backwards task and a task to stand and reach and jumping sideways, whereas boys obtained better results in a 20-meter sprint and a 6-minute run [5]. Gender differences in motor ability could be detected in the preschool years: 3- to 4-year-olds differed in the Total Motor Score, fine motor skill and a balance task, while there were no sex differences for the Total Motor Score or balance in 5- to 6-year-olds. However, 6-year-old boys outperformed girls in aiming and catching [6]. These studies suggest that gender differences in motor ability appear in early childhood; however, the direction of the obtained gender difference is rather contradictory.

In general, working memory is described as the ability to temporarily store and process information. However, more detailed models, such as the one of Baddeley [7, 8], further divide working memory into four subcomponents: the central executive, the episodic buffer, the phonological loop and the visuo-spatial sketchpad. All four components of working memory increase linearly from childhood to adolescence [9].

In contrast to visual-spatial abilities, gender exhibits rather contradicting evidence to promote working memory. A study that investigated spatial abilities by specifically examining visual-spatial working memory in a large age range of 3- to 89-year-olds indicated males slightly outperformed females in most visual-spatial working memory tasks, whereas females seemed to be favored in a task of memory for location [10]. The authors found the effect to first appear in the ages between 13 and 17. However, in an auditory working memory task females outperformed males. Gender differences in working memory also seem to be visible with respect to neuroscientific aspects. Thus, consistently more limbic and prefrontal structures are activated in females, while males showed a rather distributed activation, including parietal regions. Nevertheless, there are also studies of working memory in children that did not identify gender differences [11–13].

It is well established that the perception of the world differs with regard to the culture. One of the most relevant findings is that individuals in western countries (Americans, Western Europeans) focus on salient objects, whereas individuals in the East tend to focus on the context when remembering scenes [14]. This leads to the assumption that feature analyses of Westerners may increase the specificity of visual information contained in memory, despite equivalent memory on an item level. To date, most cross-cultural research on cognition has been performed between Asian and Western cultures, showing, for example, a superior performance in a spatial memory task of East-Asian individuals [15]. In addition, school-aged children and young adolescents in Hong Kong outperformed their United Kingdom counterparts on all four executive function tasks. Background information, such as disadvantages in scholastic or language education, may have implications for testing working memory capacity in children who grow up in poverty [16].

To date, to our best knowledge, only two experimental studies have compared cognitive abilities in Arabic and Western samples. In a sample of adults, Gudia et al. [2018] showed that spatial aspects in working memory are culture dependent. Western readers showed a left-to-right organization and Arabic readers exhibited a right-to-left organization. No spatial bias was identified for Arabic-speaking illiterates [17]. Furthermore, it was shown that in a mental rotation task, which integrates visual-spatial working memory tasks, and a cognitive processing speed task, German students outperform students from Oman [18].

Concerning the relation of motor ability and cognitive performance in general it is assumed that they are positively related throughout life, which holds true for children [19] and elderly individuals [20]. To investigate this relation, it seems important to study specific groups, such as experts in motor performance: as expected, athletes outperformed non-athletes in executive function tasks [21], processing speed [22], attention performance tasks [23] and spatial cognition. Furthermore, there has always been a particular curiosity in the relation of cognitive and motor development in children, as physical fitness has been shown to enhance information uptake and discrimination accuracy in 8- to 11-year-olds [24]. Moreover, the spatial reasoning task performance is known to determine high mathematical skills [25]. A literature review of twenty publications summarized that children with higher physical fitness and a lower BMI are more likely to perform better in school [26]. Delayed motor development has also typically be shown to correlate with cognitive deficits [27].

Regarding a possible relation of motor ability and working memory a study that investigated the relation of motor skill, mental rotation abilities and working memory in 3- to 6-year-old children showed that balance was positively correlated with different aspects of working memory [12]. Children with developmental coordination disorder showed impaired working memory functions [28]. Consisted with these findings, an afterschool physical activity program enhanced performance in tasks with greater working memory demands in preadolescent children [29]. A study that examined 7- to 10-year-old children found that eight weeks of school gymnastic training improved the response accuracy in a modified delayed match-to-sample task, and the P3 amplitude was larger in the EEG measurement [30]. Furthermore, an improvement after a one-hour recovery from a high intensity exercise training for children and adolescents (8–17 years) was found in a Digit-Span forward and backwards working memory task [31]. However, there are also contrasting studies, which indicated there was no beneficial effect of increased physical activity on working memory capacity [32]. Thus, the specific aspects of working memory (i.e., capacity or subcomponents) that relate to specific types of physical activity or motor ability must be carefully interpreted. The investigation of this relation should be of substantial interest, concerning the fact that executive functions, with working memory as one part, are crucial for school success [33] and facilitate academic achievements in adolescents [13].

The main goal of the study is to investigate the relation of motor ability and working memory performance in Omani and German children. To date, there are no data on the development of motor ability and working memory in children of the Arabic world. According to the study of Guida et al. [17], we might expect a difference between Omani and German children in different subsystems of working memory. No relevant data exist regarding potential gender differences in working memory and motoric development in both cultures at this primary school age. For the German sample, we expect a relation between motor ability and working memory ability according to the results of a younger sample [12].

## Methods

### Participants

One hundred eighty-five children, including 48 boys (mean age *M* = 8.60, *SD* = 0.61) and 42 girls (mean age *M* = 8.33, *SD* = 0.48) from Germany and 48 boys (mean age *M* = 8.00, *SD* = 0.41) and 47 girls (mean age *M* = 7.95, *SD* = 0.55) from Oman, volunteered to participate in this study. The required sample size was calculated according to the effect size in the motor development of third graders in a non-western and a western sample (d = 0.58) according to the study of Jansen et al. [2]. Given this effect size with a level of α = .05 and a probability of 1-ß = .95 a total sample size of 158 children was needed. Some more children were included because there were no relevant prior effect sizes according to possible differences in cognitive measurements of Omani and German primary school-aged children. There was a significant difference in the BMI between the Omani (*M* = 14.95, *SD* = 2.65) and German children (*M* = 17.22, *SD* = 2.77), F (1, 181) = 31.594, p < .001, partial *η2* = .149, but not between gender. All children attended third grade in a public school; for the German sample, the children were recruited from two different public schools. All children whose parents gave written informed consent for their children to participate in the study were included. None of the children suffer from any psychiatric or neurological disorder. The experiment was conducted according to the ethical guidelines of the Helsinki declaration. The study was ethical approved through the Deanship of Research at Sultan-Quaboos University, Muscat, Oman, (IG/EDU/PHED/17/02).

### Materials

The *German General Motor Test* (AST 6–11: Allgemeiner Sportmotorischer-Test) [34] for children between six and eleven years old was used to examine the motoric skills. To assess the cognitive capabilities, the Corsi Block-Tapping Test and the Digit-Span Test (forward and backwards) was applied. Because there were no standardized tests, which have been already used in research with Omani and German children the AST was chosen. This test was already successfully applied in the examination of primary school-aged children in a non-western sample [2].

### Motoric ability

The general motor test is grouped into the following skills, including speed, springiness, precision tasks and coordination:

- 20-m Run (speed task): sprint over 20 meters, best out of two runs counts
- Medicine ball (springiness exercise): long-range shot of a medicine ball (1 kg)
- Target throw (precision task): throwing a tennis ball from a 3-m distance
- Ball-Leg-Wall (precision task): throwing a gymnastics ball through the legs at the wall—turning of the body and catching it again
- Castle-Boomerang Test (whole body coordination): children had to complete an obstacle course of climbing under and jumping over lids

For a full description, refer to [2]. Scores were obtained for high precision and speed. All points were transformed into percent-ranges and z-values. In the original test of [34], a 6-Minute Run was integrated. As a result of organizational issues, this run could not be implemented in the German schools; thus, the 6-Minute Run will not be further considered. The Total Motor Score was computed by calculating the mean of the Z-scores for each child and country.

#### Working memory tests

To investigate working memory, the Corsi Block-Tapping Test [35] (forward and backwards) and the Digit-Span Test (forward and backwards) were used. The Corsi Block-Tapping Test consists of nine wooden blocks randomly assembled on a wooden board. Children must repeat each sequence typed by the experimenter in the same order (forward) or the reverse order (backwards). Remembering the sequence in the same order (forward) demands the visual-spatial sketchpad, whereas tapping the sequence backwards tests the central executive of the working memory, with a reliability of .81 and .89, respectively. The session started with a short sequence of three digits and was increased by one number if the child typed two of three sequences. This was continued until the child failed to remember three consecutive sequences. The maximum length remembered by the child was employed as the maximum score.

The Digit-Span Test (forward and backwards) is part of the Hamburger-Wechsler Intelligence Test for children [36]. Remembering the sequences in the same order (forward) measures the phonological loop, whereas repeating it in the reverse order (backwards) demands the verbal working memory processes. Children must repeat each sequence verbalized by the experimenter in the same order (forward) or the reverse order (backwards). Similar to the Corsi Block-Tapping Test, each session of the Digit-Span Test started with a short sequence of two digits. The sequence was increased by one digit until the child failed to remember both sequences of a numerical series (i.e., 2864 and 1952). The maximum length remembered by the child was employed as the maximum score. The split-half reliability for the forward task is .76; for the backward task it is.78.

### Procedure

At the beginning of each session, the demographic data, weight and height of the children were obtained. The testing started with both working memory tests, the Corsi Block-Tapping Test and the Digit-Span Test. After a break of 20 minutes, the Motoric Test was conducted. In total, the cognitive tests lasted approximately 45 minutes, and every child needed approximately 50 minutes to complete the Motoric Test.

### Statistical analysis

To investigate the effect of “gender” and “country” on the motoric abilities (20-m run, medicine ball, target throw, ball-leg-wall, and castle-boomerang test), a MANOVA was run with the five subcomponents, and a univariate analysis of variance was conducted with the Total Motor Score. Furthermore, a multivariate analysis of variance (MANOVA) was conducted with the fixed factors “group” and “gender” and four working memory aspects (Digit-Span forward and backwards and Corsi Block-Tapping Test forward and backwards). Effect sizes were given in partial *η2*. Results which were statistically highly significant were presented as *p* < .001, for all other significant results the exact p-values was given. A correlation analysis and multiple regressions were also conducted with the working memory tasks and the motoric tests. Significance levels were Bonferroni corrected, and the *p-value* was set to .0083.

## Results

### Motor abilities

Twenty of 925 data points were missing because several children did not complete parts of one task. These missing data were replaced by the respective mean of this task. The results of the MANOVA using Pillai’s trace showed an overall effect of group, *F*(5, 177) = 34.342, *p* < .001, partial *η2* = .492, but neither an effect of gender, *F*(5, 177) = 1.563, *p* = .173, partial *η2* = .042 nor an interaction between both factors, *F*(5, 177) = 1.255, *p* = .286, partial *η2* = .034. The effects for group were identified for the 20-m run, *F*(1, 181) = 86.40, *p* < .001, partial *η2* = .323, target-throw, *F*(1, 181) = 17.062, *p* < .001, partial *η2* = .086, castle-boomerang test, *F*(1, 181) = 8.445, *p* < .01, partial *η2* = .045, medicine ball, *F*(1, 181) = 18.750, *p* < .001, partial *η2* = .094 and ball-leg-wall, *F*(1, 181) = 16.589, *p* = .004, partial *η2* = .084. All values for group and gender are presented in Table 1. The Omani children scored better in the target throw and the medicine ball task, the German children in the other three tasks.

**Table 1:** Means (SD) of the motor tests.

		20-m Run	Target throw	Ball-leg-wall	Castle-Boomerang Test	Medicine ball	Total motor score
Oman	Boys	94.16 (10.77)	105.50 (10.76)	98.25 (9.82)	99.71 (9.67)	107.66 (11.00)	99.73(5.66)
	Girls	90.17 (10.23)	104.97 (11.45)	95.44 (11.29)	94.89(10.14)	107.91 (10.71)	97.95(5.44)
Germany	Boys	107.62 (13.48)	97.02 (13.88)	101.35 (10.74)	102.5(9.23)	101.35 (10.95)	101.97(8.25)
	Girls	107.76 (10.44)	99.23 (10.10)	104.19 (6.66)	100.52(10.33)	100.35 (10.81)	102.41(7.18)

The univariate analysis of variance for the z-values of the Total Motor Score showed only a main effect for group, *F*(1, 181) = 11.492, *p* = .001, partial *η2* = .060 but not for gender, *F*(1, 181) = .461, *p* = .498, partial *η2* = .003, nor a significant interaction between both factors, *F*(1, 181) = 1.273, *p* = .261, partial *η2* = .007. The performance was better for the German (M = 102.17, SD = 7.7) than the Omani children (M = 98.85, SD = 5.6).

### Cognitive ability

For the working memory tests, a MANOVA using Pillai’s trace indicated main effects of gender, *F*(4,178) = 3.024, *p* = .019, partial *η2* = .064 and group, *F*(4,178) = 30.113, *p* < .001, partial *η2* = .404; however, there was no interaction between group and gender, *F*(4,178) = 1.226, *p* = .332, partial *η2* = .027. The significant effect for gender was identified for the following cognitive tasks, the Digit-Span forward and Corsi Block-Tapping forward. Boys performed better in the Corsi Block-Tapping Test forward, *F*(1, 181) = 4.017, *p* = .047, partial *η2* = .022, and girls performed better in the Digit-Span Test forward, *F*(1, 181) = 7.541, *p* = .007, partial *η2* = .040. An effect for group was identified for all cognitive tasks, the Digit-Span forward, *F*(1, 181) = 100.46, *p* < .001, partial *η2* = .357 and backwards, *F*(1, 181) = 13.94, *p* < .001, partial *η2* = .072 and the Corsi Block Tapping Test forward, *F*(1, 181) = 6.119, *p* = .014, partial *η2* = .033 and backwards, *F*(1, 181) = 23.83, *p* < .001, partial *η2* = .116. The German children ranked better than the Omani children in all cognitive tasks, Table 2.

**Table 2:** Means (SD) of the cognitive tests.

		Digit forward	Digit backwards	Corsi Block-Tapping forward	Corsi Block-Tapping backwards
Oman	Boys	6.42 (1.22)	5.23 (1.08)	4.54 (0.68)	3.29 (0.87)
	Girls	7.17 (0.96)	5.57 (1.23)	4.47 (0.75)	3.28 (0.85)
Germany	Boys	8.79 (1.82)	6.31 (1.48)	4.98 (0.86)	3.98 (0.84)
	Girls	9.26 (1.89)	5.93 (1.42)	4.60 (0.80)	3.93 (1.16)

### Correlation-analysis of motoric skills and working memory performance

The correlation analysis between all subscales of the motor test, the Total Motor Score and the four working memory measurements indicated significant correlations between the performance in the Digit-Span forward and the 20-m Run (r = .348, p < .001), the target throw, (r = .197, p = .007), and the medicine ball, (r = .223, p = .002), and the Corsi Block Tapping Test forwards and the 20-m run (r = .206, p = .005), and the Corsi Block Tapping Test backwards and the 20-m run (r = .282, p < .001). Splitting the data for the group of Omani and German children, the two correlations did not remain significant.

## Discussion

Overall, the results indicate that compared to German children, Omani children showed a worse performance in working memory. Second, concerning motor ability, German children performed better than Omani children. There were correlations for three measurements of working memory and the ability in the 20-m run and the measurement of the Digit-Span forward and the ball throws. If the data in both countries were analyzed separately, the correlation did not remain significant.

Regarding the cultural differences in motor ability and working memory the results indicated a lower performance in the Total Motor Score of the Omani children compared to the German children. This could be explained through the fact that children in Oman do not play many games where it is important to react quickly, likely because of the high temperature or the social norms. But beside this, it is important to register that the Omani children showed a better performance in the medicine ball task as well as the target throw task. The better performance compared to German children have to be investigated in more detail. In general, children in Oman might not be used to tests of their motor ability. One of the tests, the ball-leg-wall task is an unusual posture, which the children in the Arabic culture were not accustomed to and for that the worse performance of the Omani children could be explained through social conventions. Another reason might be that physical activity is not emphasized in the Arabic culture: In adulthood, females and Arabs in general are less physically active than their respective counterparts [37]. The results could not be explained by a higher obesity rate or overweight [38] because the Omani children had a significantly lower BMI than children in Germany.

Concerning working memory, the Omani children showed a lower performance in all tasks compared to the German children. Until now, the cognitive development of children in Oman has not been investigated. Few studies are related to this research topic, one of which indicated that low birth weight in Omani children is a predictor of cognitive impairment at school age. Seventeen percent of the children born in Oman have low birth weights, and 12% of the children between 7 and 11 years perform below average in selected school marks [39]. Because this is the first study that investigated cognitive performance in Arabic compared to Western children, the reasons for the low performance may only be assumed, as the Omani children were not used to this type of testing in comparison to the German children. The cognitive measurement must be adapted to the cultural context or the children must be trained beforehand. Naturally, the concepts of the working memory tasks were explained to the Omani children; however, perhaps this should be ameliorated. Another reason for the cultural gap might be a different organization in working memory in Arabic individuals, as suggested by Guida et al. [17]. However, this would only account for the Corsi Block-Tapping Test, as the Digit-Span Test forward and backwards tasks do not investigate spatial working memory.

The gender effect for working memory shows mixed results as boys performed better in the Corsi Block-Tapping Test forward and girls performed better in the Digit-Span Test forward. Both tests were designed to retrieve information of different parts of working memory, i.e., remembering the sequences in the same order (forward) measures the phonological loop, whereas the Corsi Block-Tapping Test forward requires visual-spatial working memory. Thus, our results add to the meta-analysis of Voyer et al. [10] and show that gender differences in visual-spatial working memory are visible in primary school-aged and not, as suggested by Voyer and colleagues, first in adolescence. Furthermore, the results strengthen the findings that females have a better auditory working memory ability, which relates to the phonological loop concept of Baddeley et al. [7]. Nevertheless, the mechanisms for gender differences in working memory in primary school-aged children are far from being understood.

In contrast to other studies, the relations of motor ability and working memory were rather weak and disappeared when analyzing them separately for each culture. This finding adds to the rather contradictory literature: there was a positive correlation between balance and different aspects of working memory in preschool children [12]. Similarly, an afterschool physical activity program showed improvement in the working memory performance in preadolescent children [29, 31]. Additional studies indicated there was no beneficial effect of increased physical activity for working memory capacity. A systematic review demonstrated that weak to strong correlations were only identified for complex motor skills and higher-order cognitive skills. It also indicated that there are either no or weak correlations between the Total Motor Score and working memory [1]. The author concluded the state of knowledge as insufficient evidence for any conclusion. Thus, the relation between motor ability and aspects of cognitive performance must be discussed with caution. While the relation among fitness, inhibition, motor performance and spatial experience seemed to be evident, this is not the case for motor ability and working memory. In line with the systematic review [1] we might conclude that the relationship between motor and cognitive development only exists if the motor as well as the cognitive tasks include some kind of higher cognitive aspects. Therefore it is expected that a complex motor ability like for example movement in rhythm might enhance higher order cognitive tasks. For each intervention program this means that a complex motor skill might be best to improve cognitive performance.

From a practical point of view the results give a hint that the cognitive development of Omani children could be supported so that their performance improves to the level of children in western countries. Because working memory is a crucial cognitive function for several higher cognitive processes and also relates to academic achievement in Omani children [40], it might be worth to establish working memory training in Omani primary schools. Furthermore, due to the worse performance of Omani children in comparison to German children in the general motor score, the training of motor skill in Omani children should be ameliorated as far it is in line with cultural norms.

## Limitations and future research

This investigation is the first study to compare cognitive and motoric aspects in primary school aged children in Arabic and Western countries. The results are straightforward concerning the cultural difference; however, the mechanisms for the lower performance of the Omani children are not well understood. Further studies must integrate both the school curricula and the background of the children (education of the parents and leisure activities). Moreover, the socioeconomic status of the children in both countries must be considered because poverty in childhood is known to relate to working memory performance in adolescence [41]. Furthermore, limited aspects of cognition or executive functions were investigated; thus, it would be worth investigating potential cultural differences in switching and cognitive flexibility. Nevertheless, this is a promising approach to understand differences in the development of individuals in Arabic and German cultures.

## Supporting information

S1 Data (SAV)
